# Interleukin-37 as fundamental inhibitor of innate immunity

**DOI:** 10.1186/ar3419

**Published:** 2011-09-16

**Authors:** P Bufler

**Affiliations:** 1 Dr. von Hauner Children's Hospital, Ludwig-Maximilians-University, Munich, Germany

## 

The interleukin-1 (IL-1) family of ligands has 11 members of which most are proinflammatory. The receptors, signaling pathways, and functions of the classical family members (IL-1α, IL-1β and IL-18) have been studied extensively. However, knowledge of Interleukin-37 (IL-37/IL-1F7), which was first identified by *in silico *research in 2000 remains limited.

IL-37 shares critical amino acid residues with IL-18 and binds to the IL-18-binding protein enhancing its ability to inhibit IL-18-induced interferon-γ. Data suggest that IL-37 also binds to the IL-18Rα and, for its anti-inflammatory properties, likely recruits an accessory receptor chain with inhibitory properties, such as the single Ig IL-1 related receptor. We recently reported that overexpression of IL-37 in cells of monocytic or epithelial origin almost completely abolishes the production of proinflammatory cytokines as IL-1α/β, TNFα, IL-6 and IL-8 in response to TLR-ligands or IL-1β. Anti-inflammatory cytokines were unaffected. Vice versa, functional knockdown of IL-37 in primary human cells by siRNA increased the production of proinflammatory cytokines. IL-37tg mice are protected against LPS-induced shock. Thus IL-37 is a fundamental inhibitor of innate immune responses.

IL-37 protein is expressed in human monocytes and upregulated by LPS. Similarly to IL-1α and IL-33, IL-37 is expressed intracellularly and translocates to the nucleus upon cell stimulation in a caspase-1 dependent manner. IL-37 interacts inside the cell with Smad3 and inflammation in IL-37tg mice is increased when endogenous Smad3 is depleted. IL-37 is also secreted in the supernatant of stimulated transfected cells or peripheral mononuclear blood cells. However, the extracellular functionality of IL-37 is still elusive.

